# Ureteral distal ends combined and inserted into the ileum: a novel anastomotic technique for urinary diversion

**DOI:** 10.1186/s12894-021-00835-2

**Published:** 2021-04-19

**Authors:** Qi Wang, Liang Tang, Liangkuan Bi, Jie Min, Lu Fang, Wei Sun, Dexin Yu

**Affiliations:** Department of Urology, The Second Affiliated Hospital of Anhui Medical University, Hefei, China

## Abstract

**Background:**

This study aimed to introduce a novel method for ureteroileal anastomosis, explore its clinical effectiveness, observe the incidence of postoperative anastomotic stricture, and compare the postoperative complications with those of other types of ureteroileal anastomosis reported in the literature.

**Methods:**

Both ureters were first anastomosed at their distal ends and then inserted into the proximal end of the ileal loop. A postoperative follow-up analysis was performed to evaluate major complication indicators, including anastomotic stricture, anastomotic leak, and hydroureteronephrosis.

**Results:**

We successfully performed ureteral distal ends anastomosis combined with end-to-end insertion into the ileum for 143 patients. The mean postoperative follow-up lasted 37 months (range: 10–68 months). There was no occurrence of an anastomotic leak. The incidence of anastomotic stricture combined with hydronephrosis, ileal conduit stones, urinary tract infection, and renal dysfunction were 2.1%, 0.7%, 2.1%, and 4.2%, respectively.

**Conclusion:**

Ureteral distal ends combined and inserted into the ileum were simple to perform and helped achieve precise anastomosis with fewer postoperative complications.

## Background

Since Bricker first developed ileal conduit in 1950, it has been popularized in urinary diversion following surgical treatment of muscle-invasive bladder cancer. Given its convenience, safety, and technical maturity, it has currently become the most common method of urinary diversion for incontinent diversions [1]. Ureteroileal anastomosis is a crucial procedure in ileal conduit and a key factor affecting the quality of life and prognosis after surgery. Some patients may experience different postoperative complications, including urinary tract obstruction, urinary leakage, symptomatic infection, which are most likely related to ureteroileal anastomosis instead of cystectomy [2, 3].

There have been many methods regarding ureteroileal anastomosis in ileal conduit, including anti-refluxing or refluxing anastomosis as well as anastomotic type selection [4]. Many managements intended to reduce postoperative complications have not been improved. In recent years, our research group has improved ureteroileal anastomosis, an essential procedure in which both ureters are first anastomosed at their ends and then inserted into the ileum in an end-to-end manner. This article presented the clinical experience and efficacy of applying this technique to ileal conduit reconstruction and provided a literature review on ureteroileal anastomosis techniques and their complications.

## Methods

The present study retrospectively analyzed the clinical data of 143 patients who underwent laparoscopic radical cystectomy and ileal conduit in our hospital from 2014 to 2019. Ileal conduit reconstruction involved ureteral anastomosis at the ends of both ureters combined with end-to-end insertion into the proximal end of the ileal loop. According to the modified Clavien classification system [5], all surgical complications were recorded. The present study focused on evaluating the long-term postoperative complications after surgery, especially the incidence of anastomotic stricture and hydroureteronephrosis.

**Fig. 1:**
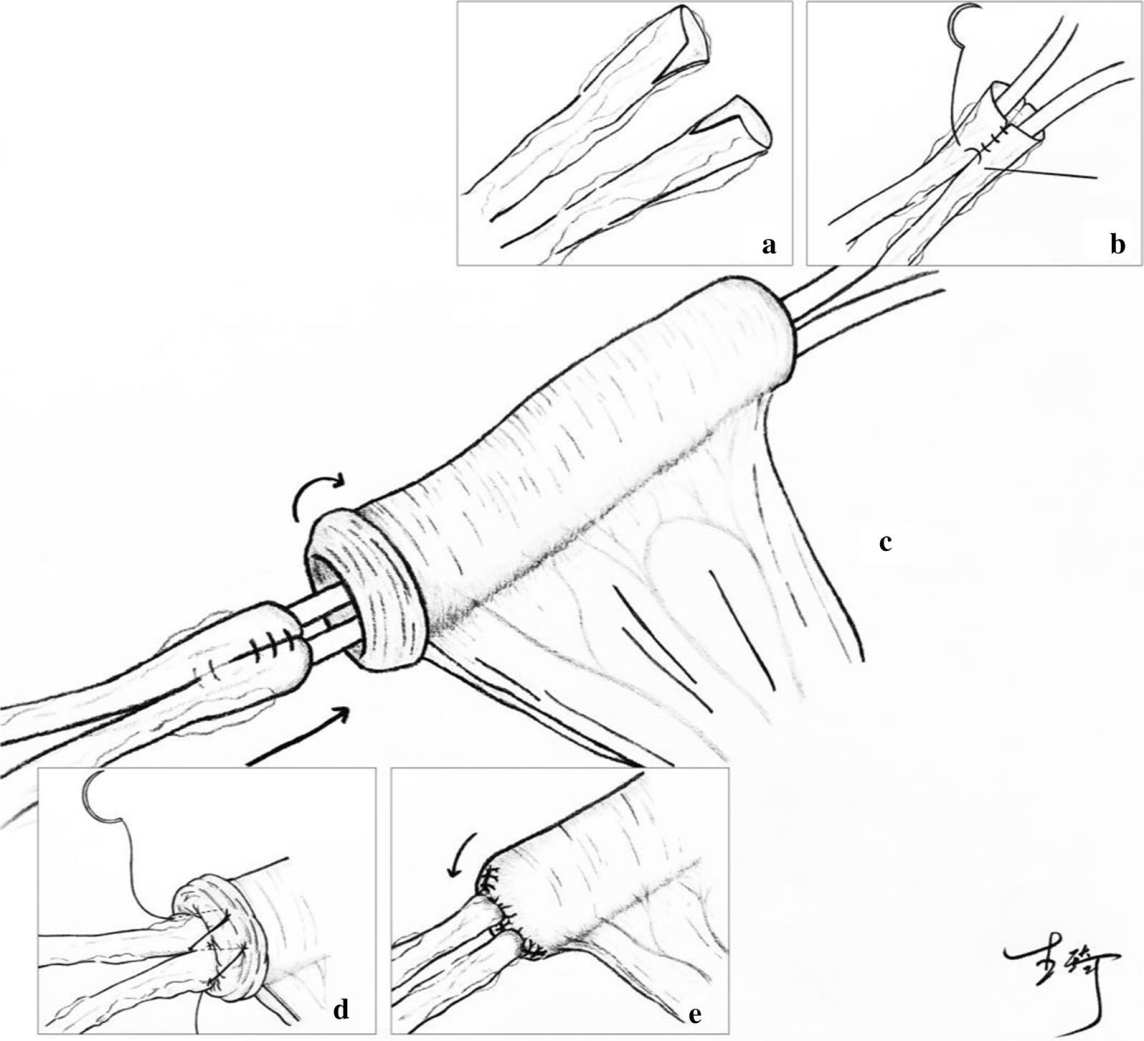
**a**, **b** The ends of both ureters were trimmed and combined. **c** A 7-Fr single J-stent was placed in each ureter and led out from the ileal loop. **d**, **e** The ureters were inserted into the ileal loop and the proximal end of the ileal loop was closed

**Fig. 2:**
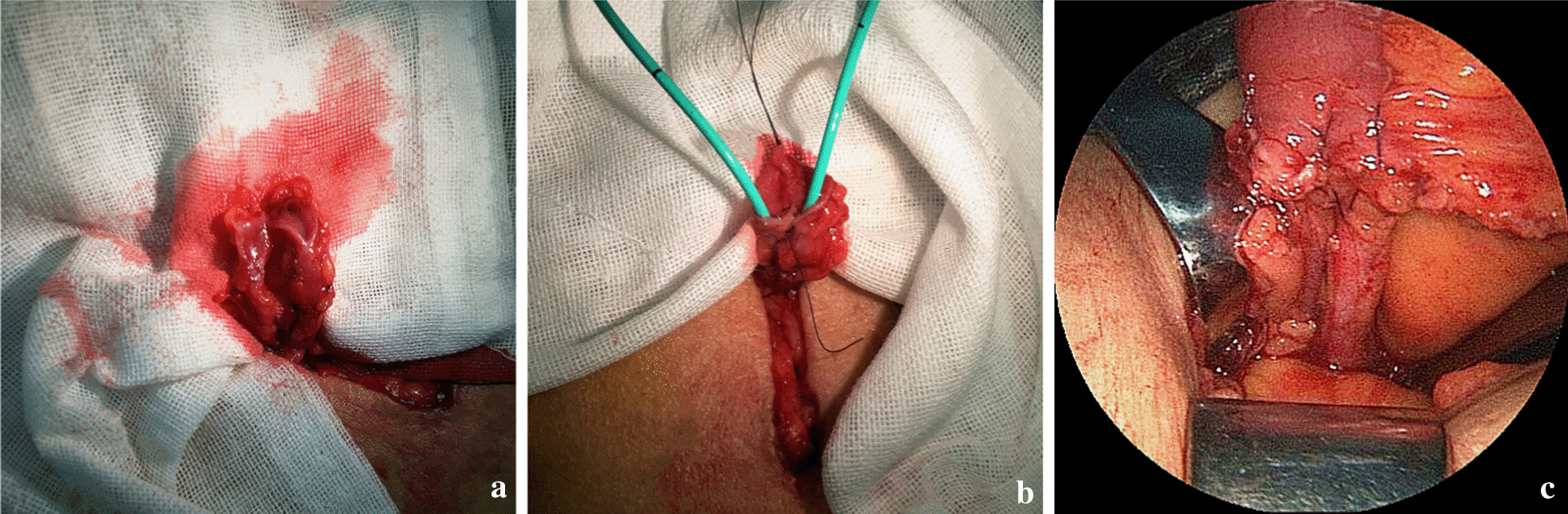
**a** Both ureters were trimmed and adipose tissue was attached to the ends. **b** After the suture of the ends of both ureters, a uniform lumen has been formed. **c** Successfully complete the ureteral anastomosis combined with end-to-end insertion into the ileum

A Urinary diversion was performed following laparoscopic radical cystectomy and pelvic lymph node dissection. First, an infraumbilical trocar incision next to the right rectus abdominis was extended longitudinally, measuring 4 cm, at which a specimen was taken out, followed by pulling the ileum out. A 10-cm-long pedicled loop of ileum was cut 15 cm away from the ileocecal region. After intestinal continuity was restored, the mesenteric hiatus was closed. The remained mesenteric length depended on the thickness of the abdominal wall to ensure adequate mesenteric blood flow. The left ureter was transposed under the mesosigmoid, pre sacredly. The distal ends of both ureters were trimmed. The nourishing blood vessels and fat surrounding the ureters were preserved as much as possible, and it was not recommended to remove too much fat attached to the ends of the ureters (Fig. 1a). The medial edge of each ureteral end was slitted longitudinally to 15 mm in length (Fig. 2a). The anterior and posterior edges of one slitted ureteral wall were separately sutured to the corresponding anterior and posterior edges of the other slitted ureteral wall with interrupted stitches using 4–0 absorbable suture, which formed a lumen. After the distal lumens of both ureters were anastomosed into one lumen, two stitches were placed on the serosal layers of the right and left ureters above the anastomotic lumen to reinforce them together, which were localized 10 mm from the upper edge of the anastomotic ureter (Figs. 1b, 2b).

A 7-Fr single J-stent was placed in each ureter and led out from the distal end of the ileal loop (Fig. 1c). The intestinal mucosa of the ileal loop's proximal end was everted like a cuff, with an eversion length of 15 mm. The distal end of the anastomotic ureter was wholly inserted into the lumen of the proximal end of the ileal loop, with the insertion length similar to the intestinal mucosa's eversion length (15 mm). Continuous stitches with a 4-0 absorbable suture were used to suture the ureteral serosa longitudinally and the ileal mucosa at the junction between the ileum and the upper edge of the inserted ureter, requiring 4–5 stitches for one circumference of the anastomosis (Fig. 1d). Deep insertion of the needle was avoided when suturing ureteral serosa, and the ureter lumen was brought slowly to the ileal mucosa upon pulling the suture. Excessive suture of the ureter tissue was avoided to prevent changes in the blood supply. Excessive traction of the ureter was avoided when pulling the suture so as to prevent ureter avulsion or ureteral wall compression. After the everted ileal mucosa was restored, continuous stitches with a 3-0 absorbable suture were used to close the proximal end of the ileal loop. When suturing around both ureters, squeezing of the intestinal wall due to excessive tightening of the suture was avoided to prevent ureter stricture (Figs. 1e, 2c). At the incision site, The ileal loop was pulled out at the incision site 4–6 cm and left outside of the body, followed by the closure of its surrounding peritoneum with a 1-0 silk suture. The external oblique aponeurosis was sutured around the lower part of the ileal loop for fixation (usually 6 stitches), which prevented retraction of the ileum. The distal end of the ileal loop formed a nipple 20 mm above the skin level, fixed with a 1-0 silk suture, completing the creation of an abdominal stoma for the ileal conduit.

## Results

A total of 143 patients were enrolled in this study, aged 63–82 years. The postoperative pathological results of all patients showed urothelial carcinoma. Pathological staging of the tumors performed using the TNM staging system. The surgery lasted ~ 187 to 263 min, and the intraoperative blood loss was ~ 50 to 280 ml (Table 1). The postoperative bowel function recovery took ~ 1 to 5 days to complete, and the patients could get out of bed ~ 1 to 3 days after the surgery. Fourteen days after the surgery, the single J-stents were removed. Postoperative hospital stays lasted ~ 5 to 13 days, with an average of 9 days.

**Table 1:** Characteristics of the studied population

Mean (SD)/Percentage (%)	
*Demographic and pathological characteristics*	
Age (years)	70.7 (14.13)
BMI, kg/m^2^, mean (SD)	23.5 (3.3)
Follow-up time, mean (SD)	37 (6.7)
Male, n (%)	106 (74.1)
Female, n (%)	37 (25.9)
*ASA score, n (%)*	
2	118 (83)
≥ 3	25 (27)
*Pathologic stage, n (%)*	
pT2	77 (54)
pT3	50 (35)
pT4	16 (11)
Operative time (min.), SD	223 (25.34)
Ureteroileal anastomosis time (min.), SD	22 (4.7)
Estimated blood loss (mL), SD	180 (66.7)

The patients whose pathology showed positive lymph nodes or staged pT3-4 began to undergo re-examination combined with adjuvant chemotherapy 1 month after surgery. The other patients were followed up from the third month after surgery. Routine follow-up items included urinary system color Doppler ultrasonography, CT and blood routine, and renal function and electrolytes, focusing on whether the patient had developed hydronephrosis (and its degree), ureteroileal anastomotic stricture, anastomotic leak, ileal conduit stones, upper urinary tract stones, urinary tract infection, and renal dysfunction.

The median follow-up time of the patients was 37 months (10–68 months). Local recurrence and metastasis occurred in 7 cases (4.9%) after surgery, and 6 died, of which 3 died of bladder tumors. Postoperative complications and treatment were (Tables 2, 3): (1) 3 cases of anastomotic stricture (2.1%), all occurring to the left side and within 12–24 months after surgery, combined with varying degrees of hydroureteronephrosis; (2) the treatment was via the ileal conduit to locate the joint opening of the ureters to enter the narrowed lumen of the left ureter for balloon dilation and insertion of a double-J stent. A postoperative review showed that the stricture was relieved, and the hydroureteronephrosis was relieved. One patient with ileal conduit stones was treated with ureteroscopic holmium laser lithotripsy, and there was no recurrence of stones in the 1-year follow-up. There were 3 patients with apparent signs of urinary tract infection: (1) 1 patient presented with anastomotic stricture, and the infection was controlled after surgical treatment; (2) 2 patients presented with no obstructive factors and no obvious hydronephrosis as confirmed with the examination. The infection was controlled with anti-infection and symptomatic treatment; (3) 6 patients presented with renal dysfunction and slightly increased creatinine levels (< 200 umol/L). Among them, 1 was treated with surgery, and 5 were given symptomatic treatment for kidney preservation, after which the renal function was improved. The reasons for early renal dysfunction may be caused by temporary hydronephrosis or transient ureteral edema after removal of single J-stents. There was no incidence of urinary anastomotic leak.

**Table 2:** Major postoperative complications in 143 patients

Anastomotic stricture (%)	Anastomotic leak (%)	Ileal conduit stones (%)	Urinary tract infection (%)	Renal dysfunction (%)
3 (2.1)	0	1 (0.7)	3 (2.1)	6 (4.2)

**Table 3:** Treatment of postoperative complications

No	Complications	Time from cystectomy to diagnosis	Treatment
1	Anastomotic stricture Urinary tract infection Renal dysfunction	12 mo	Balloon dilation and insertion of a double-J stent + anti-infective therapy
2	Anastomotic stricture	15 mo	Balloon dilation and insertion of a double-J stent
3	Anastomotic stricture	20 mo	Balloon dilation and insertion of a double-J stent
4	Ileal conduit stone	10 mo	URSL + Insertion of a double-J stent
5	Urinary tract infection	7 mo	anti-infective therapy
6	Urinary tract infection	3 mo	anti-infective therapy
7	Renal dysfunction	5 mo	Protection of renal function
8	Renal dysfunction	3 mo	Protection of renal function
9	Renal dysfunction	24 mo	Protection of renal function
10	Renal dysfunction	18 mo	Protection of renal function
11	Renal dysfunction	34 mo	Protection of renal function

## Discussion

Radical cystectomy combined with urinary diversion is the primary surgical method for treating muscle-invasive bladder tumors, among which urinary diversion has a decisive impact on the quality of life of patients after surgery. Ileal conduit surgery is the gold standard procedure for incontinent diversions. For patients who are not suitable for orthotopic neobladder reconstruction, the ileal conduit is the first choice. Due to its short surgical duration and simple formation steps, the ileal conduit is more suitable for patients with more serious underlying diseases and elderly patients [6–8].

Whether the patients present with postoperative renal ureteral dilatation, urinary leakage, or symptomatic upper urinary tract infections such as pyelonephritis, the root cause is ureteroileal anastomotic stricture or anastomotic leak. Such ureteroileal anastomosis-related complications are most common, accounting for 25%-60% of all complications [9, 10]. The most common methods for anastomotic construction are: (1) the end-to-side ureteroileal anastomosis (Bricker anastomosis), which generally involves inserting the ureteral ends through the sidewall of the ileum into the intestinal segment to a depth of about 1 cm and then suturing around the insertion site. Therefore, the anastomotic stoma is prone to angulation, the inserted ureter has a short free length, and scars from around the anastomotic stoma; (2) the end-to-end ureteroileal anastomosis (Wallace anastomosis), which involves slitting the medial wall of the distal end of each ureter for approximately 3 cm, laying both ureters adjacent to each other, suturing the two medial edges to form a single lumen, and anastomosing the lateral edges of the single lumen to the ileal end. This procedure is prone to cause the excessive free length of the lower ureters, which reduces the distal blood flow, causes an excessive anastomotic tension, and results in an extensive anastomosis range. Before the formation of an ileal conduit in these two surgical procedures, the free length of the middle and lower part of the left ureter (which was moved to the right) should be longer than that of the right ureter, thereby resulting in a higher degree of traction injuries and blood flow decrease, as well as a higher suture tension. Therefore, stricture and urinary leakage are relatively common in the left ureteroileal anastomosis [4, 11, 12].

The documentary on applying different ureteroileal anastomosis techniques in recent years was reviewed, focusing on anastomotic leak and stricture compared with the present results (Table 4). Despite modifications, the Bricker anastomosis is still prone to anastomotic stricture, while the Wallace anastomosis is more likely to cause anastomotic leak [5, 9, 11, 13]. The present anastomosis procedure led to a significantly lower incidence of anastomotic stricture (2.1%) than the two anastomosis techniques above and did not result in anastomotic leak in any patient.

**Table 4:** Summary of literature reporting late complications related to ureteroileal anastomosis in bladder cancer patients after ileal conduit surgery

Reference	Technique	Total (cases)	Anastomotic leak	Anastomotic stricture
Christoph et al. [11]	Bricker	75	–	19 (25.3)
	Wallace	65	–	5 (7.7)
Kavaric et al. [5]	Modified Wallace	70	6 (8.5)	2 (3)
Li et al. [9]	Modified Bricker	145	4 (2.8)	5 (3.4)
The present study	Combined with end-to-end insertion	143	0 (0)	3 (2.1)

**Fig. 3:**
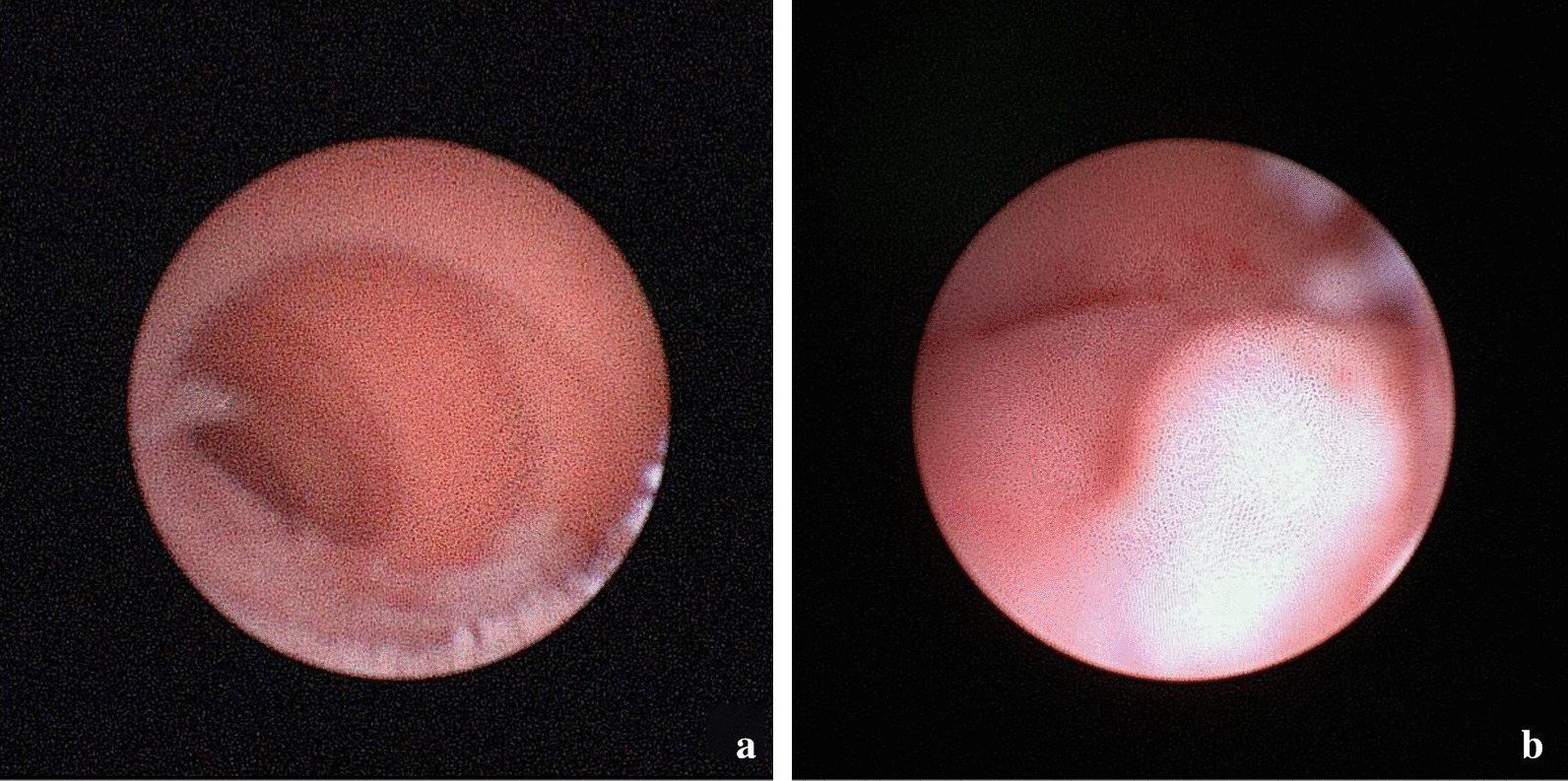
**a**, **b** The ureteroscopy entered the ileal conduit, and the common opening of bilateral ureters was found at its proximal end

During the ureter and ileum's anastomosis, the ends of both ureters were slitted, reconstructed, and sutured together to form a single lumen. Next, the reshaped lumen was entirely inserted into the proximal end of the ileal loop with an insertion length of about 1.5 cm. The ureteral serosa and the ileal mucosa at the junction between the ileum and the upper edge of the inserted lumen were anastomosed, and the proximal end of the ileal loop was closed. This anastomosis procedure avoided two problems: (1) the stricture, angulation, and concealment of ureteral anastomosis due to large amounts of ileal mucosal folds in the immediate proximity of the ureteral anastomosis; (2) the difficulty of locating the opening under direct vision in later re-examination and thus the failure to explore the ureters. The advantages of ureteral anastomosis combined with end-to-end insertion into the ileum were mainly manifested in four aspects. (1) The longitudinal slitting and reconstruction of the distal ends of both ureters enlarged the ureteral diameter and eliminated the contraction of the circular muscle in the ureteral wall, minimizing the incidence of luminal stricture. The slit length was only 15 mm, and thus this method was superior to the Wallace method in protecting the distal blood vessels. (2) Anastomotic stricture is generally attributed to the scar tissues formed at the anastomosis site. At the same time, there was no direct anastomosis between the distal opening of the conjoined ureters and the ileal mucosa and no contact between the distal end and the ileal mucosal folds, thereby avoiding the stricture and obstruction at the distal ureteral opening that would otherwise be present due to surgical scars and fold occlusion. (3) In the end-to-end insertion mode, the free length was long without anastomosis angulation, and there was no apparent high-tension anastomosis during surgery, so the anastomosis was more exact, thereby reducing the probability of urinary leakage. (4) Even in postoperative anastomotic stricture, it was easy to locate the joint distal opening of both ureters at the proximal end of the ileal conduit under a ureteroscope and explore each ureter to locate the narrowed segment and conduct treatment accordingly (Fig. 3a, b). Such a procedure is safe and convenient, even applicable in the case of urinary tract stones. It should be noted that the key to the successful implementation of the ureteral anastomosis combined with end-to-end insertion into the ileum is to maintain sufficient blood flow when freeing the ureters and to ensure that the anastomosis is performed in the absence of significant tension. Otherwise, ureteral insertion into the ileum can result in a certain degree of tension and affect the distal blood flow, which in turn can increase the incidence of postoperative anastomotic leak and stricture.

This anastomosis method allows the ureters and the ileum to be conjoined in their respective physiological peristaltic directions so that the peristaltic path of the ureter and the ileum are consistent without noticeable deviation and angulation, thereby forming a peristaltic flow to promote urinary excretion [14]. The normal peristalsis of the ureters after surgery can stimulate and drive the ileal peristalsis to a certain extent, which effectively removes the intestinal mucus produced by the goblet cells in the intestinal wall thereby reducing the risk of intestinal mucus accumulation obstructing the urethral opening. Moreover, the normal peristalsis facilitates timely and thorough drainage of urine in the ileal conduit into an ostomy bag, reducing the residence time of bacteria in the ileal conduit, and avoiding prolonged contact of urine with mucus due to poor drainage and thereby inflammatory changes, which in turn reduces the incidence of retrograde upper urinary tract infection after surgery [15].

As a retrospective clinical study, the present study had certain limitations, especially the absence of a control group for comparison. More research is needed to prove further the feasibility of this technique based on more clinical data. However, the present results have shown that this technique is safe and effective. As indicated by the follow-up data, the present anastomosis technique has better clinical outcomes than the traditional techniques, achieving a significantly lower incidence of anastomotic stricture and urinary leakage as the primary complications.

## Conclusion

Ureteral distal ends combined with end-to-end insertion into the ileum is more favorable for reducing the incidence of postoperative anastomotic stricture, anastomotic leak, and urinary tract infection. It is also convenient to use for dealing with postoperative complications such as hydroureteronephrosis, ureteral stricture, and stone formation, thereby making it worthy of wide clinical application. Considering that there is no clinical standard for ureteroileal anastomosis, further studies are needed to explore better anastomosis methods for the patients.

## Data Availability

The datasets used and/or analysed during the current study are available from the corresponding author on reasonable request.
